# The root transcriptome for North American ginseng assembled and profiled across seasonal development

**DOI:** 10.1186/1471-2164-14-564

**Published:** 2013-08-19

**Authors:** Di Wu, Ryan S Austin, Sijun Zhou, Dan Brown

**Affiliations:** 1 Western University, 1151 Richmond St, London, ON, Canada; 2 Agriculture and Agri-Food Canada, 1391 Sandford Street, London, ON, Canada; 3 Canadian Centre for Agri-food Research in Health and Medicine (CCARM), St. Boniface Hospital, 351 Taché Avenue, Winnipeg, MN, Canada

**Keywords:** North American ginseng, Transcriptome, Next generation sequencing, Ginsenoside

## Abstract

**Background:**

Ginseng including North American ginseng (*Panax quinquefolius* L.) is one of the most widely used medicinal plants. Its success is thought to be due to a diverse collection of ginsenosides that serve as its major bioactive compounds. However, few genomic resources exist and the details concerning its various biosynthetic pathways remain poorly understood. As the root is the primary tissue harvested commercially for ginsenosides, next generation sequencing was applied to the characterization and assembly of the root transcriptome throughout seasonal development. Transcripts showing homology to ginsenoside biosynthesis enzymes were profiled in greater detail.

**Results:**

RNA extracts from root samples from seven development stages of North American ginseng were subjected to 454 sequencing, filtered for quality and used in the *de novo* assembly of a collective root reference transcriptome consisting of 41,623 transcripts. Annotation efforts using a number of public databases resulted in detailed annotation information for 34,801 (84%) transcripts. In addition, 3,955 genes were assigned to metabolic pathways using the Kyoto Encyclopedia of Genes and Genomes. Among our results, we found all of the known enzymes involved in the ginsenoside backbone biosynthesis and used co-expression analysis to identify a number of candidate sequences involved in the latter stages ginsenoside biosynthesis pathway. Transcript profiles suggest ginsenoside biosynthesis occurs at distinct stages of development.

**Conclusions:**

The assembly generated provides a comprehensive annotated reference for future transcriptomic study of North American ginseng. A collection of putative ginsenoside biosynthesis genes were identified and candidate genes predicted from the lesser understood downstream stages of biosynthesis. Transcript expression profiles across seasonal development suggest a primary dammarane-type ginsenoside biosynthesis occurs just prior to plant senescence, with secondary ginsenoside production occurring throughout development. Data from the study provide a valuable resource for conducting future ginsenoside biosynthesis research in this important medicinal plant.

## Background

Ginseng belongs to species within the genus *Panax* (family Araliaceae) that comprises approximately 14 species of slow-growing perennial plants with fleshy roots [1]. North American ginseng (*Panax quinquefolius* L.) is native to eastern North America [2] and *Panax* species have been used for thousands of years to improve the overall health of human beings, as a remedy to promote vitality, assist the body functions, improve the immune system and protect against stress. It also has been recommended for years as a traditional medicine for a multitude of diseases such as cancer, inflammation, diabetes, cardiovascular ills and obesity [3-7] as well as being a recent source of natural extracts mass marketed as a cold prevention treatment [8].

Ginsenosides are considered to be the major bioactive compounds behind many of the claims of ginseng’s health benefits; they are triterpenoid saponins found nearly exclusively in ginseng and have been the target of considerable research effort [9-11]. To date, more than 150 naturally occurring ginsenosides have been isolated from *Panax* species and most of them can be classified into two groups based on the skeleton of their aglycones, namely dammarane-type and oleanane-type [12,13]. The dammarane-type consists mainly of three varieties, classified according to their genuine aglycone moieties: 20S-protopanaxadiol (PPD), 20S-protopanaxatriol (PPT), and ocotillol. Rb1, Rb2, Rc, Rd (PPDs) and Re and Rg1 (PPTs) are the most abundant six ginsenosides found in North American ginseng. Over 90% of total ginsenoside content from North American ginseng belongs to these two groups [14,15].

North American ginseng contains high levels of Rb1, Rd and Re ginsenosides—higher than those of *Panax ginseng*[16]. Ro is the only saponin of the oleanane-type ginsenoside, found as a minor component in North American ginseng [14]. Ginsenosides are biosynthesized via the mevalonate pathway [17]. Using expressed sequence tag analysis, it has been possible to identify several candidate genes encoding for the enzymes farnesyl diphosphate synthase (FPS) and squalene synthase (SQS), involved in the various biosynthetic steps from isopentenyl pyrophosphate and dimethylallyl pyrophosphate to squalene [18-20]. The cyclization of oxidosqualene is the branch point for the biosynthesis of ginsenosides and plant sterols. The common steps from acetyle-CoA to 2, 3-oxidosqualene have been widely studied [21]. The 2, 3-oxidosqualene cyclases (OSCs) that synthesize β-amyrin and dammarenediol-II [22,23], as well as the Cyt P450 enzyme CYP716A47 that catalyses the formation of protopanaxadiol from dammarenediol-II during ginsenoside biosynthesis have been found in *Panax ginseng*[24]. However, the rest of the downstream pathways of ginsenoside biosynthesis remain largely unexplored.

Over the past several years, next-generation sequencing technologies have revolutionized the analysis of genomic information [25]. As applied to the transcriptome with RNAseq, it has been successfully used for transcript profiling, as well as SNP discovery in a number of plant species and has dramatically improved the efficiency and speed of gene discovery [26-28]. The application of 454 next generation sequencing technology has seen a rapid improvement in throughput, read length and accuracy in the past few years, with the GS FLX Titanium server used in this study able to generate one million reads with an average length of 400 bases with 99.5% accuracy [29]. Meanwhile, the analysis of transcriptomic data often relies on aligning reads to a reference which is often not feasible for non-model plant species in which little genomic research has been performed. We applied the transcriptome assembly program Trinity to the assembly of a high quality reference transcriptome for North American ginseng. Trinity has been shown to recover most expressed transcripts as full-length sequences, and is also able to resolve alternative isoforms and duplicated genes, outperforming other *de novo* assembly tools [30,31]. Our application to ginseng resulted in 41,623 ginseng root transcripts. We fully annotated 84% of these transcripts using sequence similarity searches and protein domain scanning with publicly available databases. In our results, we were able to identify predicted representatives for all of the known enzymes involved in the ginsenoside backbone biosynthesis and also profile their expression levels across seasonal development.

## Results

**Figure 1:**
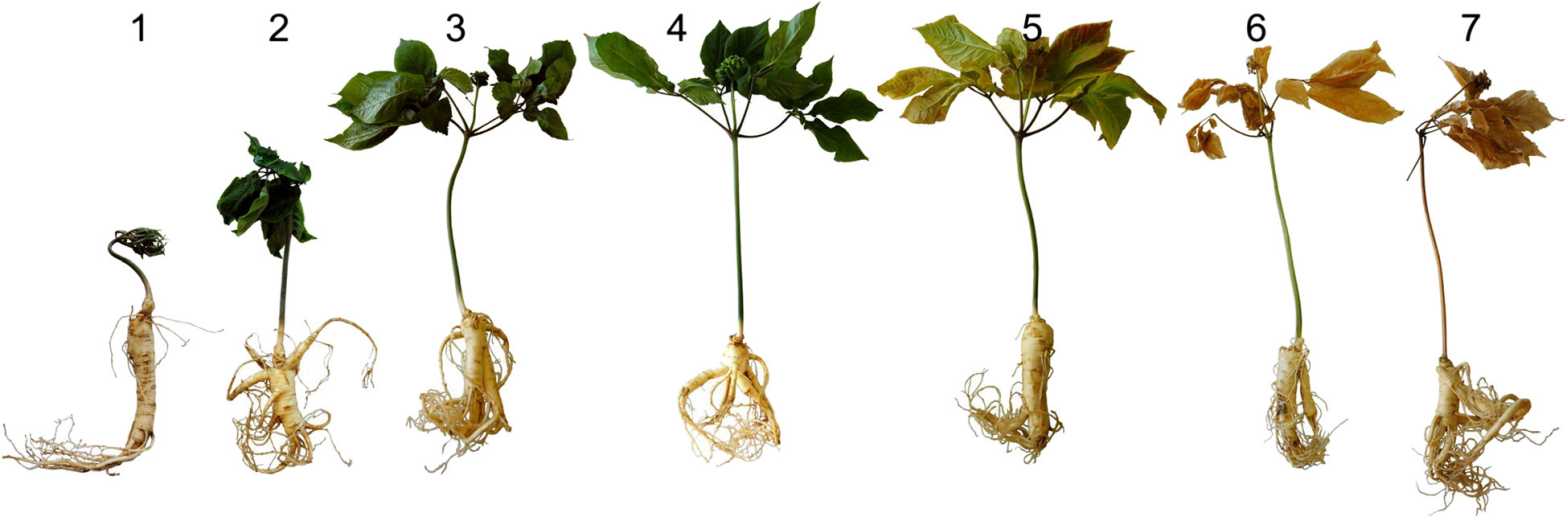
**North American ginseng seasonal development stages used for harvest. 1)** budding - leaf emergence; **2)** leaves - distinctive separation of leaf and stem; **3)** flowering - plant in full flower; **4)** green fruit set; **5)** ripe fruit - fruit coloration fully red; **6)** fruit drop – including early signs of leaf senescence (e.g., leaf curl) and **7)** senescence - complete senescence of leaf and stem (the normal commercial harvest stage).

Commercial production of ginseng usually results in harvests after 3 to 5 years. In this study, three-year-old roots were collected, washed, sorted for uniformity and overwintered and grown under simulated growing season conditions in the Biotron facility to minimize variation in environmental factors and soil pathogen infection. Root samples were collected over the fourth-year full growing season at seven development stages [32]: 1) budding - leaf emergence; 2) leaves - distinctive separation of leaf and stem; 3) flowering - plant in full flower; 4) green fruit set; 5) ripe fruit - fruit coloration fully red; 6) fruit drop – including early signs of leaf senescence (e.g., leaf curl) and 7) senescence - complete senescence of leaf and stem (the normal commercial harvest stage) (Figure 1).

### Sequencing and *de novo* assembly

Tissue from the seven developing stages was collected from 4-year-old North American ginseng grown under controlled conditions in a growth chamber programmed to simulate southern Ontario, Canada commercial growing conditions (Additional file 1). Ginsenoside content levels for each development stage in American ginseng are listed in Table 1. Total RNA was isolated from the roots of each stage, and messenger RNA purified by Ambion Poly (A) Purist™ mRNA Purification Kits. A half plate of 454 sequencing on the GS FLX Titanium platform was applied to each sample (http://www.454.com). This generated from 327 to 391 Mbp of sequence for each stage with an average sequence length of 553 base pairs. All sequencing reads from the seven development stages were deposited in the NCBI (National Centre for Biotechnology Information) and can be accessed at the Sequencing Read Archive (SRA) with the accession numbers SRX247045, SRX247043, SRX247042, SRX247040, SRX247039, SRX247038 and SRX247037 for stages one through seven respectively.

**Table 1:** **Ginsenoside contents of development stage among American ginseng ( *P *. *quinquefolius*) in Ontario**

	**Ginsenoside composition**^**a **^**(ug/g, 75% Ethanol extraction)**						**Total ginsenosides (mg/g)**
**Stage**	**Rg1**	**Re**	**Rb1**	**Rc**	**Rb**	**Rd**	
1	2251	30602	91611	7879	1072	15795	14.92
2	1441	22318	116252	11024	2104	17338	17.05
3	1273	19313	86478	7669	1185	15194	13.11
4	2395	22863	82086	6042	965	11751	12.61
5	1755	22408	73849	6452	994	7324	11.28
6	2055	22268	85416	5970	964	6999	12.37
7	1671	15019	94093	6083	1000	8523	9.11

a: Samples were tested in duplicate and number of root samples tested in each stage.

**Figure 2:**
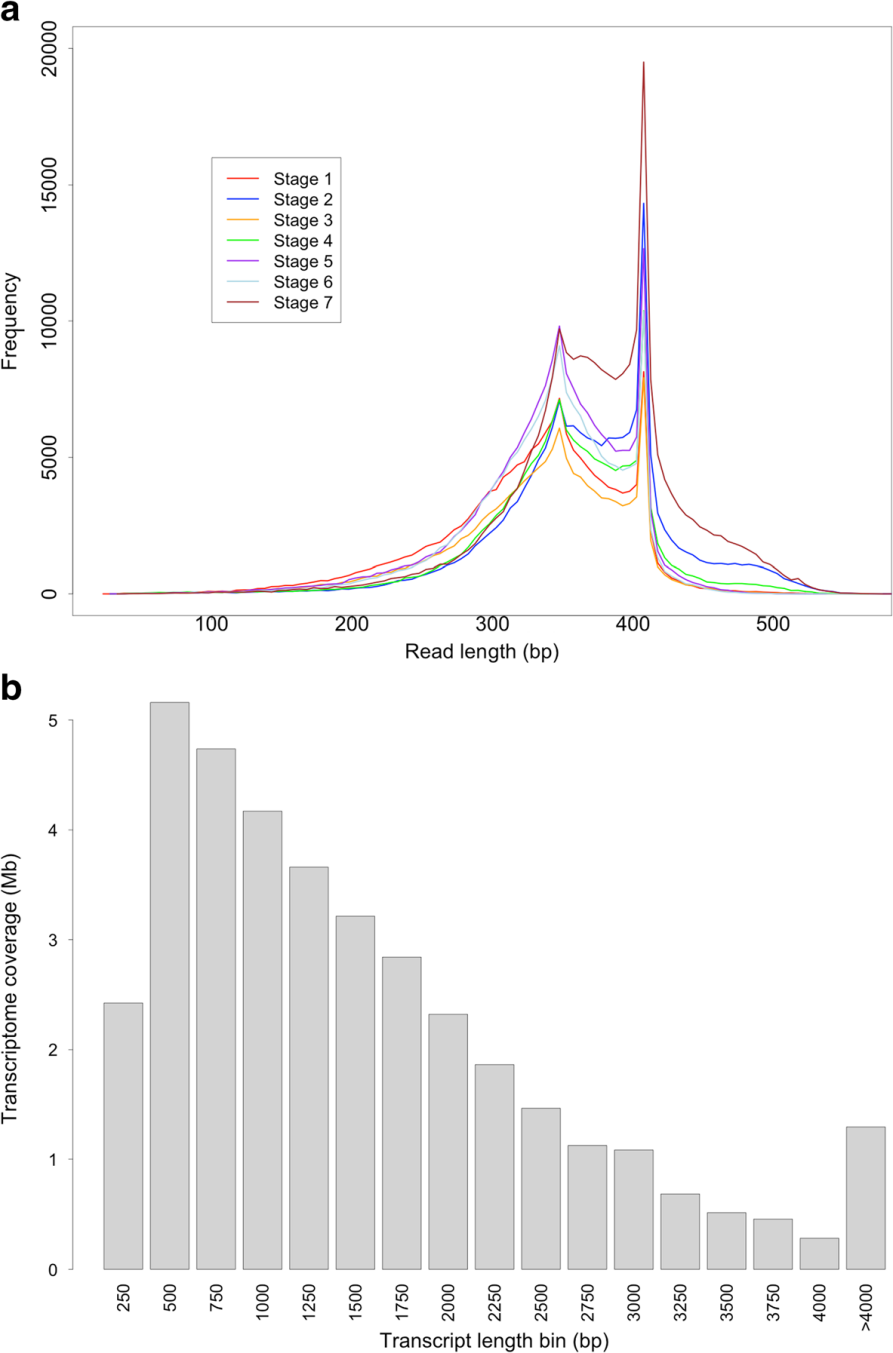
**Filtered reads and assembled transcript length distributions. (a)** Distribution of sequence read lengths used in transcriptome assembly from each of the seven developmental stages after quality filtering and trimming. **(b)** Histogram of assembled transcripts binned at 300 bp intervals and the relative contribution of each bin to the overall transcriptome size.

Filtering of the sequence data before assembly included steps that removed plastid contaminants and adaptor sequences, as well as trimmed the base pair bias present in the first 15 bp of the 5’ end and low quality bases (Q < 30) at the 3’ end. Quality score distributions for reads in each stage before and after filtering are provided in Additional file 2. After filtering, 1,222,382 sequence reads remained for assembly with an average length of 348 base pairs (Table 2). Unfortunately, our poly-A purification step failed to effectively filter the abundant rRNA present and a large percentage of reads had to be filtered from each stage as contaminants arising from ribosomal or plastid RNA sequences. Interestingly, after quality trimming the reads, a disproportionate amount of sequences with lengths of approximately 340 and 410 base pairs were found (Figure 2a). This is presumably an artefact of trends in sequence quality drop off at specific points during sequencing.

**Table 2:** Summary of cDNA sequencing results

	**Stage 1**	**Stage 2**	**Stage 3**	**Stage 4**	**Stage 5**	**Stage 6**	**Stage 7**
No. raw reads	748,332	610,853	712,270	672,867	721,897	756,630	625,784
Raw nt (Mb)	390.6	327.8	372.2	361	368.4	388.2	327.2
Raw avg. length (bp)	522	537	728	537	510	513	523
No. filtered reads	159,812	173,693	132,038	149,483	191,274	173,273	242,359
Filtered nt (Mb)	52.6	63.9	44.1	52.7	65.3	59.0	90.5
Filt. avg. length (bp)	329	368	334	353	341	340	373
% in assembly	58.5	62.6	62.3	64.2	60.1	62.0	61.2

**Figure 3:**
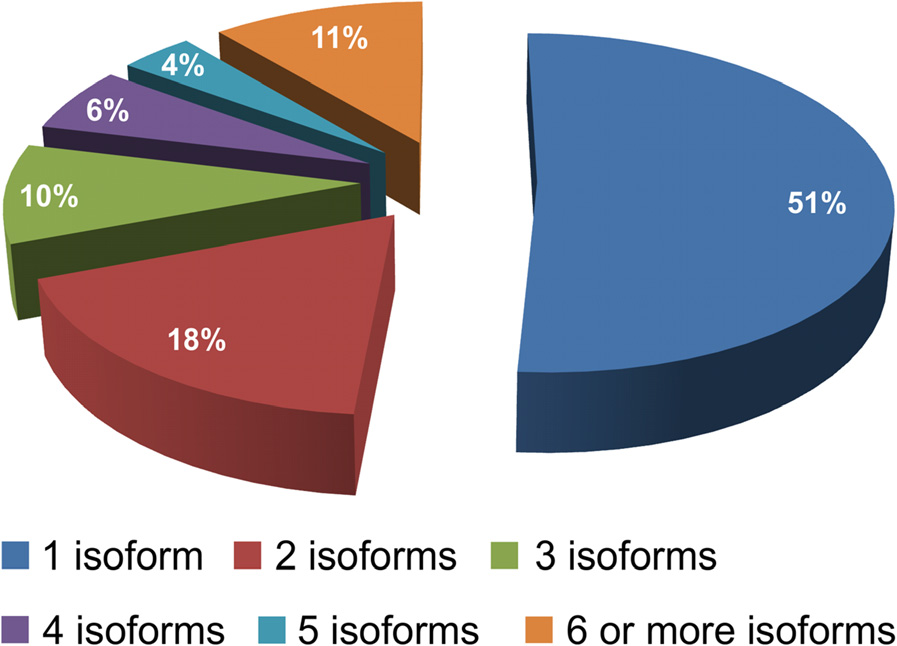
**Isoform abundance in the assembled transcriptome.** The percentage of the assembly possessing splice variants rang from a single transcript to 6 or more isoforms for a single gene.

High quality reads from all stages were combined and provided to the transcriptome assembly program Trinity. The resulting assembled 37.3 Mb of transcriptome contained 21,340 genes or 41,623 transcripts when including the different gene isoforms Trinity is capable of returning. This number represents more than 8 times the number of North American ginseng sequences presently deposited in Genbank (as of March, 2013). Transcript lengths ranged from 300 to 7,719 base pairs with an average length of 896 bp and the majority of transcripts ranging between 500 bp and 2Kb in size (Figure 2b). Almost half of all genes assembled (49%) possessed at least one isoform, with a total of 20,283 splice variants identified by Trinity and 11% of genes possessing 6 or more splice variants (Figure 3). One gene (Pq315) possessed 96 different isoforms, although we felt this may have been an artefact of the assembly process.

In a similarity comparison to 5,018 *Panax quinquefolius* ESTs in Genbank, 87.82% were present in our assembly with strong significance (E < e^-20^). When Genbank ESTs specifically derived from the *Panax quinquefolius* rhizome were considered, this number increased to 92.66%, suggesting a high quality, comprehensive sampling of the root developmental transcriptome. To simplify identification and enable easy reference, all sequences in the assembly were assigned a unique identifier derived from the Trinity graph component and appended with a splice number that followed the form of “Pqx.y”, where “Pq” stands for *Panax quinquefolias*, “x” is the Trinity component number and “y” is the splice variant number.

### Transcript annotation with public databases

To facilitate as complete an annotation as possible for the assembly, sequence similarity searches [33] were performed against a collection 5,018 Ginseng ESTs from GenBank, the *Arabidopsis* genome (TAIR10), the uniProt Plant Protein Annotation Program (PPAP) database and GenBank’s non-redundant (nr) protein database. In addition, protein domain scanning using hidden Markov models (HMMs) from Pfam were applied as well as the assignment of metabolic pathway information from the Kyoto Encyclopedia of Genes and Genomes (KEGG) database. Overall, these efforts annotated 83.6% of transcripts in the assembly.

Comparison against the Genbank nr database yielded 33,366 hits (E < 1e^-10^), accounting for 80.16% of transcriptome sequences. A full 87.2% of these hits showed strong homology (E < e^-20^). As the nr database contains few ginseng proteins, we examined the different species with which homology was found. The majority of hits (55.7%) were found to be against grape (*Vitis vinifera*), followed by castor oil plant (*Ricinus communis*) (14.5%), black cottonwood poplar (*Populus trichocarpa*) (12.4%), and soybean (*Glycine max*) (1.6%). Similar results were found with searches against the Plant Protein Annotation Program (PPAP) database from Uniprot and the TAIR10 release of the *Arabidopsis* genome yielding 33,522 (80.54%) and 30,990 (74.45%) transcripts with significant hits (E < 1e^-10^) (Additional file 3).

**Figure 4:**
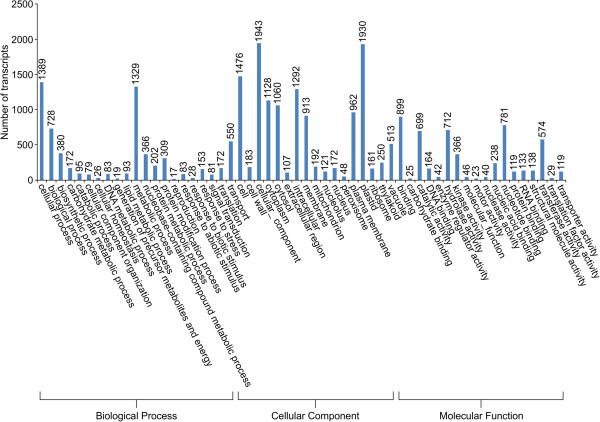
**Functional classification of North American ginseng based on gene ontology categories.** Gene ontology categories and classifications as identified by feeding the assembled transcriptome to the BLAST2GO web server.

As *Arabidopsis* is the most thoroughly annotated plant, sequence homology to *Arabidopsis* was also used to characterize the transcriptome based on Gene Ontology (GO) information. GO annotation provides descriptions of gene products in terms of their associated molecular functions, cellular components, and biological processes. Using sequence homology to TAIR10, 14,537 GO terms were assigned to 24,110 sequences categorized into 80 functional groups. GO assignments were most frequently related to biological processes (6,431), followed by cellular components (12,489) and molecular function (5,190) (Figure 4).

The assembly was scanned with protein domain HMM models from the Pfam database in order to catalogue any significant matches (E < e^-10^) to known protein domains. Overall, 32,277 HMMs were scanned against the assembly resulting in annotation for 21,263 transcripts possessing 17,266 different protein domains. This added annotation information to an additional 77 transcripts that had no hits in the prior homology searches. The most abundant domain found was the protein kinase domain, present in 1,310 transcripts. This is a similar number to the 1,719 kinases present in the *Arabidopsis* genome and not surprising, as protein kinases play a role in a multitude of cellular processes, including division, proliferation, apoptosis, and differentiation.

Finally, in order to assign metabolic information to our transcripts, the KAAS tool (http://www.genome.jp/tools/kaas/)[34] was used to assign pathway information from the KEGG database. This resulted in a KEGG orthology (KO) number for 5,717 transcripts that possessed homology with metabolic enzymes in the KEGG database. The pathways most strongly represented in the results were protein processing in the endoplasmic reticulum, ubiquitin mediated proteolysis, nucleotide excision repair, arginine and proline metabolism, peroxisome, amino sugar and nucleotide sugar metabolism, proteasome, starch and sucrose metabolism and glycolysis/gluconeogenesis. All annotation information and significance scores were summarized and concatenated with transcript identifiers into a single line of annotation for each sequence in a Fasta formatted transcriptome file (Additional file 3).

### Expression profiles across development

To evaluate the contribution of each stage of development to the reference transcriptome and profile the relative expression levels of transcripts throughout development, we mapped all high quality reads from each stage back to the assembled transcriptome using the BWA tool [35]. While this is useful in determining relative transcript abundance, it did have the unfortunate side effect of reporting unmapped reads for 6,113 transcripts. Investigating further, it was found that nearly all unmapped transcripts were involved in alternative splicing and of the shortest transcripts in the assembly (mean length: 414 bp). We presume that this is a consequence of the k-mer (k = 25) assembly process used by Trinity, perhaps creating isoform transcripts that are shorter than the long 454 reads from which they originally derive or possibly from read trimming by the alignment program. Nevertheless, expression information was obtained for over 85% of the transcriptome which comprised the longer contigs.

Counts for mapped reads were then used to generate RPKM (reads per kilobase transcript per million reads mapped) values for each putative transcript in each developmental stage. Of the most abundantly expressed predicted proteins, storage proteins topped the list, with a number of *Panax ginseng* annotated proteins: RNase-like major storage protein, specific abundant protein, tonoplast intrinsic protein, major latex-like protein and dehydrin 4 (Table 3).

**Table 3:** **Top 20 most highly expressed transcripts in *P*. *quinquefolius *root transcriptome evaluated across all developmental stages**

**Unique ID**	**RPKM**	**Function**
Pq00.2	50250.3	Ribonuclease-like storage protein [*Panax ginseng*]
Pq00.1	34703.4	Ribonuclease-like storage protein [*Panax ginseng*]
Pq250.4	14090.4	Specific abundant protein-like protein 1 [*Panax quinquefolius*]
Pq230.7	9875.59	S-adenosylmethionine synthetase 2 [*Arabidopsis thaliana*]
Pq411.1	9742.28	Tonoplast intrinsic protein [*Panax ginseng*]
Pq291.2	8594.7	Methionine synthase [*Hemp broomrape*]
Pq420.1	8176.25	Major latex-like protein [*Panax ginseng*]
Pq480.1	7957.75	Ribosomal protein L15 [*Populus balsamifera subsp*. *trichocarpa*]
Pq380.1	7692.05	tRNA/rRNA methyltransferase (SpoU) family protein [*Arabidopsis thaliana*]
Pq540.1	7655.38	Thioredoxin H-type 1 [A*rabidopsis thaliana*]
Pq140.1	7191.77	Plasma membrane intrinsic protein PIP1-1 [*Fraxinus excelsior*]
Pq323.5	7188.64	Ascorbate peroxidase [*Nicotiana tabacum*]
Pq140.3	6558.44	Aquaporin [*Iris hollandica*]
Pq470.11	6270.28	Actin 7 [*Arabidopsis thaliana*]
Pq430.1	6038.08	Translation initiation factor SUI1 family protein [*Arabidopsis thaliana*]
Pq660.2	6003.21	Nucleic acid-binding, OB-fold-like protein [*Arabidopsis thaliana*]
Pq93.7	5982.31	Dehydrin 4 [*Panax ginseng*]
Pq740.1	5947.93	β-amylase 3 [*Arabidopsis thaliana*]
Pq400.1	5940.15	DCD domain protein [*Arabidopsis thaliana*]
Pq571.2	5831.33	Catalase 2 [*Arabidopsis thaliana*]

**Figure 5:**
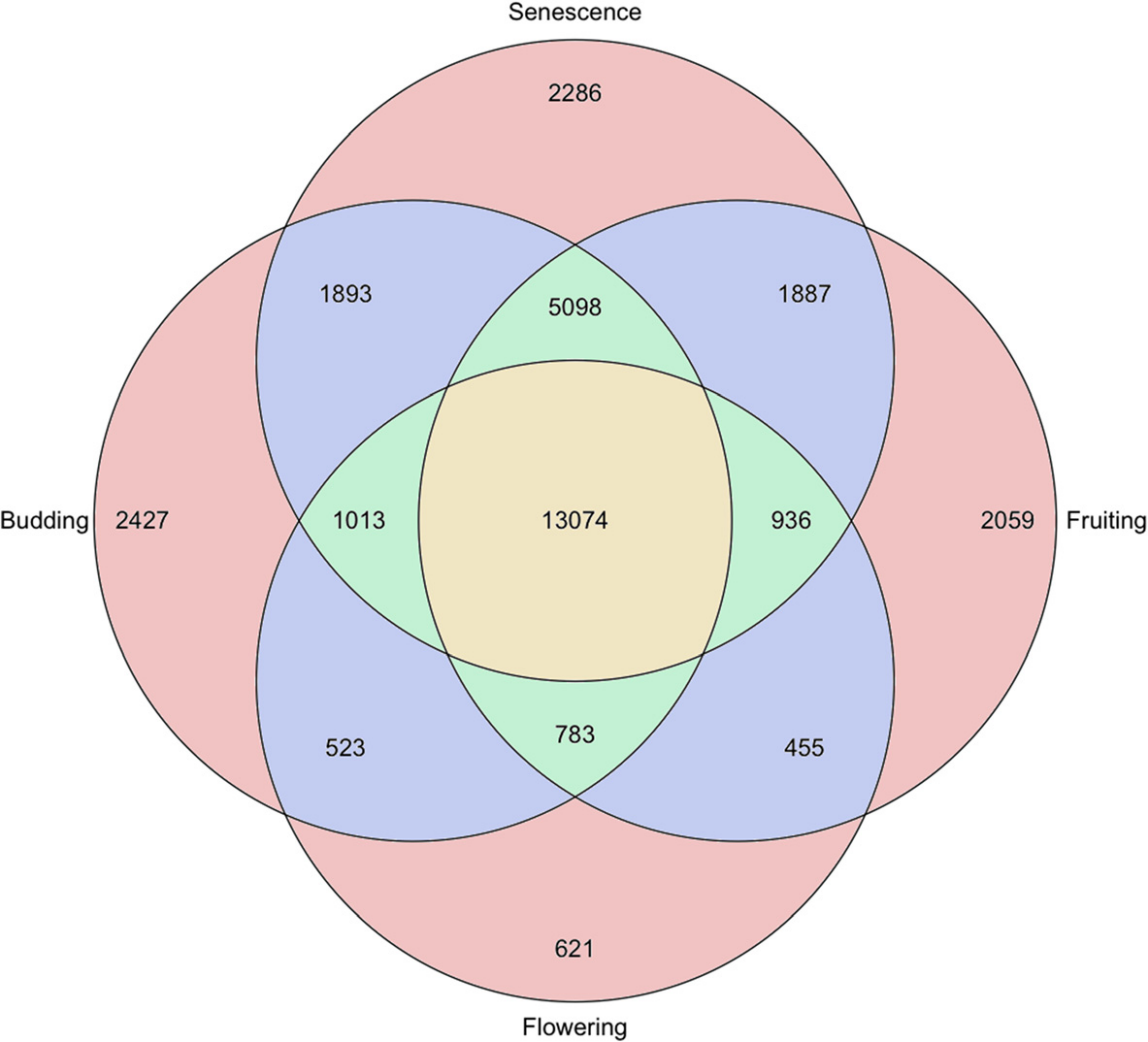
**Venn diagram of transcript abundance across development.** The 7 developmental stages sampled were grouped into four categories for a simplified comparison of transcript abundance: Budding – stages 1 & 2; Flowering – stage 3; Fruiting – stages 4 & 5; Senescence – stages 6 & 7. RPKM values for transcript expression in each developmental category were used to establish transcript presence or absence and results compared.

We also examined the level of overlap in transcript expression occurring between major developmental stages of the plant. To limit the complexity in the number of comparisons needed, the seven developmental stages sampled were grouped into the broader developmental categories of budding (stages 1/2), flowering (stage 3), fruiting (stages 4/5) and senescence (stages 6/7). Overall, 26,681, 17,990, 26,162 and 26,772 predicted transcripts showed positive expression in each category respectively. Each category possessed around two thousand predicted transcripts uniquely expressed during that stage of root development, with the exception of flowering, which had 621 specific transcripts. However, this is likely influenced by the fact that flowering is the only single stage group and thus represents a snapshot from a shorter period of developmental time compared to the other stages. Interestingly, the senescence stage had the largest number of predicted transcripts overlapping with other developmental stages. Altogether, a total of 13,074 transcripts were found to be expressed throughout all stages of development (Figure 5).

As ginsenosides are the ultimate compound of commercial interest in ginseng harvest, we sought to inventory all putative transcripts in the assembly that could be implicated in the synthesis of ginsenosides and examine their expression profiles across seasonal development. We therefore took all KEGG orthology numbers associated with enzymes in the mevalonate pathway of terpenoid backbone biosynthesis along with those from the sesquiterpenoid and triterpenoid biosynthesis pathways leading to chair-chair-chair-boat conformation triterpenoids (of which ginsenosides belong) and pulled all transcripts annotated with corresponding KO numbers in the assembly. This returned 14 predicted genes (43 transcripts including isoforms) annotated and identified by KEGG orthology. In addition, as there was no appropriate assigned KEGG EC for β-amyrin synthase, five β-amyrin synthase annotated genes (6 transcripts including isoforms) were pulled for analysis using homology annotation in the assembly (Table 4).

**Table 4:** Assembly genes implicated in ginsenosides biosynthesis

**Gene**	**Alias**	**EC**	**KO**	**Annotation**
Pq9090	AACT	2.3.1.9	K00626	Acetyl-CoA acetyltransferase
Pq24050	HMGS	2.3.3.10	K01641	HMG-CoA synthase
Pq28460	HMGR	1.1.1.34	K00021	HMG-CoA reductase (NADPH)
Pq48060	MK	2.7.1.36	K00869	Mevalonate kinase
Pq106070	PMK	2.7.4.2	K00938	Phosphomevalonate kinase, mvaK2
Pq40790	MDD	4.1.1.33	K01597	Mevalonate diphosphate decarboxylase
Pq10790	IDI	5.3.3.2	K01823	Isopentenyl-diphosphate-isomerase
Pq57960	FPS/DMAPP	2.5.1.1	K14066	Farnesyl diphosphate synthase/IPP-dimethylallyltransferase
Pq25790	FPS/DMAPP	2.5.1.1.1/10	K00787	Farnesyl diphosphate synthase/IPP-dimethylallyltransferase
Pq130580	FPS/DMAPP	2.5.1.1.1/10	K13789	Farnesyl diphosphate synthase/IPP-dimethylallyltransferase
Pq44950	FPS/DMAPP	2.5.1.1.1/10	K13789	Farnesyl diphosphate synthase/IPP-dimethylallyltransferase
Pq28280	SQS	2.5.1.21	K00801	Squalene synthase
Pq129610	SQE1	1.14.13.132	K00511	Squalene epoxidase 1
Pq7190	SQE1	1.14.13.132	K00511	Squalene epoxidase 1
Pq41740	DS	4.2.1.125	K15817	Dammarenediol-II synthase
Pq137240	AS			β-amyrin synthase
Pq133900	AS			β-amyrin synthase
Pq215750	AS			β-amyrin synthase
Pq104900	AS			β-amyrin synthase
Pq194830	AS			β-amyrin synthase
Pq75200	P450			Cytochrome P450, CYP73A5
Pq7360	P450			Cytochrome P450, CYP71B25
Pq9780	P450			Cytochrome P450, CYP75B1
Pq10460	P450			Cytochrome P450, CYP71A26
Pq142430	P450			Cytochrome P450
Pq108220	P450			Cytochrome P450, CYP710A1
Pq137680	GT			UDP-Glycosyltransferase
Pq47050	GT			Glycosyltransferase, GAUT9
Pq177130	GT			Glycosyltransferase, GAUT14
Pq117050	GT			UDP-Glycosyltransferase
Pq158770	GT			α 1,4-Glycosyltransferase
Pq10160	GT			UDP-Glycosyltransferase, 71C4
Pq315*	GT			UDP-Glycosyltransferase

* This gene possessed 96 splice variants and may be an artefact of assembly.

Squalene synthase plays an important role as the precursor in backbone biosynthesis of the dammarenediol-type ginsenosides [36,37]. There are three previously reported squalene synthases in *Panax ginseng*[38] including squalene synthase (PgSS1), squalene synthase 1 (SQS1) and squalene synthase 2 (SQS2). SQS1 and SQS2 were also previously found in *Panax quinquefolius*[39]. Although, we found 3 putative genes (i.e. Pq71210, Pq96200, Pq28280) with a significant squalene/phytoene synthase domain (Pfam: PF00494), only one (Pq28280) was annotated with KEGG orthology to SQS. This gene showed strong identity (E = 0.0) with *A*. *thaliana* the SQS1 gene.

In North American ginseng, the majority of ginsenosides are known to be of the dammarene-type ginsenosides produced from protopanaxdiol and protopanaxtriol triterpenes. Dammarenediol-II produces protopanaxdiol and protopanaxtriol, and ginsenosides are thought to be synthesized from subsequent hydroxylation of these products by cytochrome P450 enzymes and glycosylation by glycosyltransferases (GTs) [22-24]. Our assembly contained 175 predicted transcripts annotated as Cytochrome P450s; with 63 of these possessing high similarity to P450 sequences from *Panax ginseng* and *Panax notoginseng* EST collections. Similarly, the assembly contained 164 predicted transcripts annotated as glycosyltransferases with 54 of these previously identified in *Panax ginseng*, *Panax notoginseng* and *Panax quinquefolias*. In order to identify potential candidates from these gene families that may be functioning in the latter stages of ginsenoside biosynthesis, we conducted a co-expression analysis with the transcript profiles for our putative dammarenediol synthase (DS) and squalene expoxidase (SQE) found immediately upstream in the ginsenoside biosynthesis pathway.

The expression profiles for the two strongest SQE (Pq129610.1) and DS (Pq41740.1) annotated transcripts in our assembly showed very high co-expression (r > 0.89). This is not unexpected given that DS follows SQE in the biosynthesis pathway. We reasoned that candidate downstream P450 and glycosyltransferase genes may be similarly co-expressed. Co-expression analysis between all putative P450s and our predicted DS transcript identified 6 candidate P450s highly co-expressed with DS (r > 0.85) across the 7 developmental stages sampled (Pq75200.2, Pq7360.1, Pq9780.1, Pq10460.1, Pq142430.1 and Pq108220.1). In the case of Pq75200.2, co-expression with DS was extremely high (r > 0.96). Similarly, 6 glycosyltransferases annotated transcripts were highly co-expressed with our predicted DS (r > 0.85) (Pq137680.1, Pq47050.2, Pq177130.1, Pq117050.1, Pq158770.1, and Pq10160.3). As before, one transcript (Pq137680.1) showed extremely high co-expression (r > 0.94). Similar results were found in co-expression with the upstream SQE (data not shown). It should be noted that two splice variants from a single gene were also returned in the glycosyltransferase co-expression analysis. However, these transcripts derived from a gene (Pq315) for which 96 splice variants were identified by Trinity. As the annotation information for these 96 isoforms showed no strong consistency across isoforms, we concluded that this may be an artefact of the assembly, perhaps from the misinterpretation of a gene family, and were therefore dropped to simplify the analysis.

**Figure 6:**
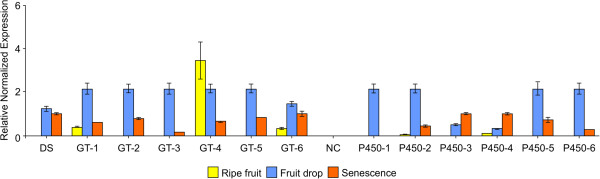
**Real time PCR analysis.** Real-time PCR results for 6 putative cytochrome P450 transcripts and 6 putative glycosyltransferase transcripts found to be highly co expressed (r > 0.9) with a predicted dammarenediol synthase (DS) evaluated across the last 3 development stages of ripe fruit, fruit drop and senescence. Corresponding unique transcript identifier numbers in the assembly are: DS: Pq41740.1; GT-1: Pq137680.1; GT-2: Pq177130.1; GT-3: Pq117050.1; GT-4: Pq158770.1; GT-5: Pq47050.2; GT-6: Pq10160.3; NC - negative control; P450-1: Pq75200.2; P450-2: Pq7360.1; P450-3: Pq9780.1; P450-4: Pq10460.1; P450-5: Pq142430.1; P450-6: Pq108220.1.

In order to provide biological validation to the 12 predicted transcripts showing coexpression with our putative DS gene, we conducted real-time PCR against the DS gene itself, the 6 putative glycosyltransferase genes and 6 putative cytochrome P450 genes across the final 3 stages of development. We were able to successfully amplify all 13 genes using RT-PCR analysis and confirmed strong coexpression between our predicted DS, 5 of the glycosyltransferase transcripts and 4 of the P450 transcripts (Figure 6). Additional file 4 lists the primers used in this analysis.

**Figure 7:**
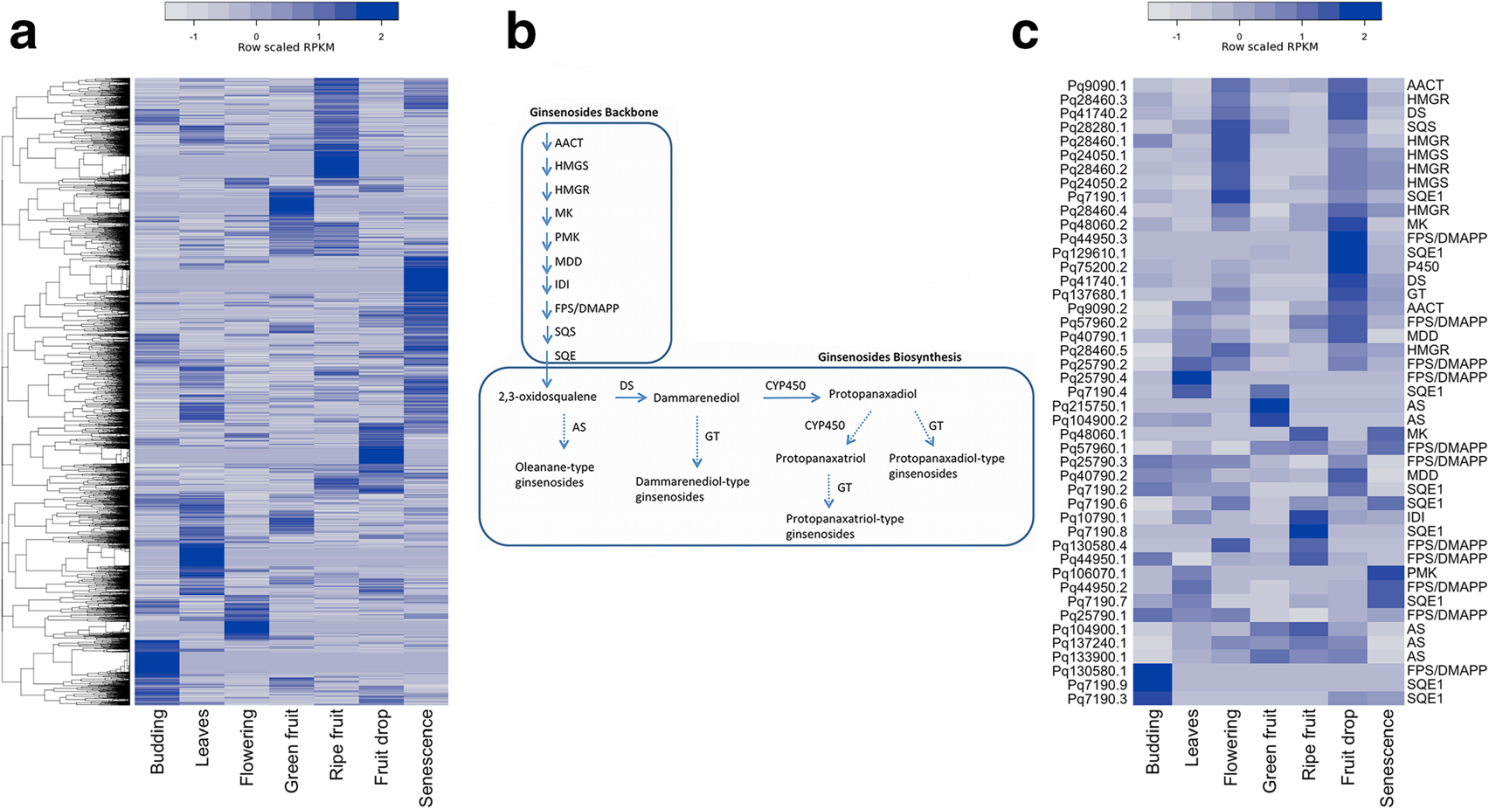
**Putative ginsenoside biosynthetic pathway. (a)** Row-wise scaled RPKM values across seven developmental stages for all transcripts in the assembly, hierarchically clustered and colour coded by increasing relative expression. **(b)** The ginsenoside biosynthesis pathway: AACT, acetyl-CoA acetyltransferase; AS, β-amyrin synthase; DMAPP, IPP-dimethylallyltransferase; DS, dammarenediol-II synthase; FPS, farnesyl diphosphate synthase; GT, glycosyltransferase; HMGR, HMG-CoA reductase; HMGS, HMG-CoA synthase; IDI, isopentenyl diphosphate isomerase; MDD, mevalonate diphosphate decarboxylase; MK, mevalonate kinase; P450, cytochrome P450; PMK, phosphomevalonate kinase; SQE, squalene epoxidase; and SQS, squalene synthase. **(c)** Heat map of all identified ginsenoside biosynthesis genes in the assembly hierarchically clustered and mapped using row-wise scaled RPKM values.

Before examining the relative expression profiles of our identified biosynthesis genes we sought to first look at the expression profiles for the entire transcriptome across seasonal development. RPKM values for all predicted transcripts were thus hierarchically clustered, and displayed in a heat map to create a transcriptome-wide display of developmental expression (Figure 7a). Many transcripts showed maximum expression within one or two specific stages of root development relative to other stages, with all stages of development possessing distinct clusters of genes showing dominant expression within that stage. Moreover, compared to other stages of development, senescence possessed a disproportionately large cluster of transcripts exhibiting a senescence-specific maximum in expression.

Expression profiles for all predicted genes and their isoforms implicated in ginsenoside biosynthesis (Figure 7b) as identified above were also hierarchically clustered and plotted in a heat map (Figure 7c). For representatives of downstream candidates, the most highly co-expressed P450 and glycosyltransferase transcripts identified in the co-expression analysis were also included in the collection. As seen with the entire transcriptome, many enzymes displayed abundant expression, specific to one or a few developmental stages relative to the other stages. To our surprise, stage 3 (Flowering) and stage 6 (Fruit drop) both possessed obvious clusters that encompassed putative representatives for almost all the enzymes in the biosynthesis pathway, suggesting that these two developmental stages are important points of ginsenoside biosynthesis in the developing plant. In the case of stage 7, this makes intuitive sense considering the traditional time of ginseng harvest after the fruit drop stage and onset of leaf senescence. Interestingly, when different isoforms of genes were present, they tended to exhibit expression maxima in different stages of development. This was most apparent with the 9 different isoforms of SQE1 (Pq7190), for which at least one instance of an isoform with stage specific expression was found in all developmental stages except senescence stage 7 (Figure 7c). Of course, due to the computational limitations of assembly and mapping, both the isoforms identified and the expression profiles should be interpreted with caution without further *in planta* validation.

## Discussion

Ginsenosides are found exclusively in the plant genus *Panax* and are viewed as the primary active compounds behind the claims of ginseng's health efficacy. The ginsenosides, also known as triterpenoids belong to the class of terpenoid saponines and are synthesized via the mevalonate pathway, which shares a common pathway with sterol. Oxidosqualene is a precursor common to the biosynthesis of both steroid and triterpenoids in higher plants [36]. The cyclization of 2, 3-oxidosqualene to oleanane and dammarane triterpenes is the first step in ginsenosides synthesis. The primary enzymes of triterpenoid saponions synthases are β-amyrin synthase (AS), and lupeol or dammarenediol-II synthase (DS). Putative orthologs for DS and AS were both identified in our assembly. β-amyrin synthase synthesizes oleanane-type triterpene and oleanane-type ginsenosides; however, they are found in only minor amounts in North American ginseng. On the contrary, over 90% of the total ginsenosides of North American ginseng belong to the dammarane-type. Interestingly, in our results, predicted AS transcripts appear most abundantly within developmental stages in which predicted DS enzymes are not abundant (i.e., flowering and fruiting). Moreover, they are not associated with the distinctive co-expression clusters of abundant putative ginsenoside biosynthesis genes as seen with DS in stages 3 and 6. This potentially emphasizes the minor role of AS derived ginsenosides in North American ginseng. At the same time, the clusters of predicted biosynthesis enzymes during flowering and fruit drop highlight a potential significance of damamarane-type synthesis and suggest that flowering and fruit drop could be primary points of ginsenoside biosynthesis during seasonal development, with most emphasis on the latter.

While we observed distinct stages of specific up-regulation for most predicted enzymes in the assembly, the presence of different isoforms with expression abundance specific to different stages of development is intriguing. In particular, our putative SQE1 possessed 9 different isoforms that showed stage specific expression abundance in all stages of development save stage 6 (fruit drop). Curiously, an alternate predicted SQE1 (Pq129610.1) with only a single isoform was strongly expressed in this stage. This seems to suggest that variation in alternative splicing may be a mechanism for producing varied forms of ginsenosides during seasonal development. Generally speaking, it also implies that alternative splicing may function as a means for directing variation in secondary metabolite production throughout the course of plant development. However, due to the inherent computational limitations involved in assembly and mapping, further analysis in the form of qPCR and associated metabolic assays is needed to prove or disprove any such hypotheses.

Of course, the assembly process will not be perfect with respect to isoform prediction and the transcripts themselves. There is a strong potential for misassembly in the form of merged gene families, close paralogs, or even alleles of the same gene being misreported as isoforms. While the comparison with Ginseng ESTs in Genbank is reassuring of the assembly quality, all predictions should be treated with caution in the absence of biological validation. Similarly, the mapping of reads to the assembly is limited by the presence of isoforms, as the actual point of origin for the read is confounded by the presence of potential multiple sources. This introduces a level of stochastic noise to the expression analysis that is mostly confined to genes with several isoforms.

That said, real-time PCR was able to validate the presence of a number of transcripts within expected developmental stages, as well as confirm their coexpression and upregulation within the fruit drop stage of development. Transcripts for a predicted DS gene, six putative P450s and six putative glycosyltransferases were all confirmed as present *in planta* and expression levels for four of the P450s and five of the glycosyltransferases confirm a tight coregulation with the predicted DS gene across the last stages of development as seen in our expression data. These predicted enzymes are thus strong candidates for controlling ginsenoside biosynthesis in the late stages of plant development.

While this study, as any next-generation sequencing study, would have benefited from an even larger amount of sequence data, additional sequencing over so many stages of development was unfortunately cost prohibitive. Nevertheless, the overall structure of the assembly with regard to number of genes, isoform frequency, length of transcripts and level of homology with existing EST libraries and annotation patterns identified among the transcripts is all very supportive and indicative of a strong representation of the biology. Overall, we believe the analysis benefited considerably from the use of the much longer reads that 454 sequencing is capable of generating. This added information translates into more reliable, longer, and complete transcripts, as well as more information for improved accuracy in the calling of alternative splicing among sequenced transcripts.

Recent work by Sun et al., found similar benefits to the use of 454 sequencing in the assembly of a transcriptome for North American ginseng [39]. Our work has expanded on these initial efforts through the generation of over six times the amount of high quality 454 reads sampled over seven stages of seasonal root development. This provides an unprecedented snapshot of North American ginseng root development and a means to comprehensively capture root expressed transcripts and their relative abundance through developmental time. In addition, we have also taken advantage of the recent Trinity assembly algorithm, which is designed specifically for assembling transcriptomes. Altogether, this provides the benefit of a high confidence, reliable assembly of putative transcripts that includes potentially valuable splice variant information. In the end, the combination of a fully annotated assembly along with corresponding expression data as sampled across developmental time has provided for an insightful look into the transcriptome of North American ginseng.

## Conclusions

Ginseng is a valuable medicinal herb that is widely used in traditional medicine and has considerable commercial value. This study has focused on the ginseng root as it is the tissue almost exclusively used for medicinal applications and contains the majority of the commercially and medicinally valuable ginsenosides. Using extensive sampling of root tissue across seasonal development and next-generation sequencing we have generated a reliable North American ginseng transcriptome with extensive annotation and isoform information. Expression profiling of all ginsenoside backbone biosynthesis genes suggests that ginsenoside biosynthesis is tightly linked with the developmental stages of flowering and fruit drop. We anticipate the assembly and associated expression data should prove invaluable to those conducting transcriptomic and metabolomic research against *Panax quinquefolius*.

## Methods

### Sample collection and preparation

In the first week of October, three-year-old North American ginseng (*Panax quinquefolius* L.) was collected from the field of a cooperating commercial grower and member of the Ontario Ginseng Growers Association (http://ginsengontario.com/), near Delhi, Ontario, Canada. Plants were washed, sorted for size uniformity and placed in a peat-based growing medium (Promix BX, Priemer Tech Horticulture) filled containers in the Biotron facility at Western University (http://www.thebiotron.ca/). After a simulated over-wintering treatment at 4°C in the dark for 120 days, plants were transferred to a growth chamber in the first week of February under conditions which simulated the normal spring / summer field conditions for temperature, sunlight and humidity in the southern Ontario commercial growing region (Additional file 1). The four-year-old roots were collected at seven development stages (Figure 1), rinsed with cold water, chopped into small pieces, and immediately immersed in liquid nitrogen and stored at -80°C until further processing.

The possibility of transcript “contamination” by bacterial and fungal pathogens was minimised by careful cleaning of the roots and growth in a peat-based commercial (Promix BX) growing medium. This medium contained a biostimulate (*Bacillus pumilus*) and mycorrhizae (*Glomus intradices*). Pathogens commonly found in Canada colonised on *Panax* roots [40] include: *Phytophthora cactorum*, *Pythium ultimum*, *Rhizoctonia solani*, *Fusarium solani*, *F*. *oxysporum*, *F*. *aveaceum*, *F*. *equiseti* and *Cylindrocarpon destructons*. A search of the final assembly annotation after blasting against the NR database showed no hits against these species.

### RNA extraction

One gram of frozen root tissue was ground to a fine powder in liquid nitrogen and transferred into 10 ml RNAzol® RT reagent (Molecular Research Center, Inc). This was vortexed vigorously for 5 min to make a complete suspension before 4 ml RNase free water was added, incubated for 15 min (r.t.), 2 ml bromo-chloroform (Sigma) added and centrifuged at 4°C 12,000 rpm (14,900 g) for 15 min. The supernatant was transferred into a new tube, 10 ml phenol chloroform (25:24:1, pH6.8, Ambion) added, mixed, and centrifuged at 4°C 12,000 rpm (14900 g) for 15 min. The supernatant was transferred to a new tube and 3 ml isopropanol plus 3 ml 1.2 M NaCl added to precipitate total RNA. The mixture was incubated 15 min (r.t.), spun at 12000 rpm (14,900 g) for 15 min at 4°C, and the supernatant discarded. 10 ml of 75% ethanol was added to the pellet, vortexed to mix and then centrifuged for 10 min at 8,000 rpm (6630 g). The supernatant was discarded and the pellet resuspended with 3 ml nuclease free water (65°C). An equal volume of phenol: chloroform (25:24:1, pH 6.8) was added, the mixture vortexed and then centrifuged for 15 min at 12,000 rpm (14,900 g). The supernatant was mixed with 0.1 volume 5 M ammonium acetate and 2.5 volumes 100% ethanol. This was placed at –20 C overnight, or quickly frozen in either ethanol or dry ice, or in a –80°C freezer for 30 min. RNA was recovered by centrifugation at ≥12,000 × g for 30 min at 4°C. 1 mL of 70% ethanol was added to the pellet and the tube vortexed. The RNA was then pelleted by microcentrifugation at 12,000 rpm (14,900 g) for 10 min at 4°C and the pellet dissolved in 50 ul nuclease free water. Extracted total RNA was cleaned using an RNeasy Mini Kit (QIAGEN).

### Sample preparation and sequencing

Total RNA preparations were poly-A enriched prior to sequencing using a Poly (A) purist^TM^ magnetic mRNA purification kit (Ambion). Isolated mRNA was qualified and quantified using an Agilent RNA 6000 pico kit on an Agilent 2000 Bioanalyser. Approximately 600-800 ng of isolated mRNA of each sample was sent to the DNA Technologies Laboratory at the National Research Council Canada for analysis. Samples were converted into cDNA using a cDNA Rapid Library Preparation Method (Roche) and sequenced on a GS FLX sequencer.

### Assembly and annotation

Sequencing reads were filtered for contaminating plastid and ribosomal RNA sequences by comparison of all reads with a file of potential contaminants using BLAST (E < 1e ^-10^). Custom Perl scripts were then used to remove any adaptor sequences, a base pair bias artefact from sequencing present in the first 15 bp of the 5’ end and low quality bases (Q < 30) at the 3’ end. Filtered reads from all stages were concatenated together and fed to the Trinity assembler with a k-mer length of 25 and minimum transcript length of 300 bp. Similarity searches for annotating transcripts were performed using the BLAST blastn algorithm against Ginseng ESTs from Genbank, UniProt PPAP and TAIR10_pep_20101214_updated databases, and the blastx algorithm against Genbank nr. The Plant Protein Annotation Program (PPAP) database was built from the concatenation of the ‘sprot’ and ‘trembl’ files for plants downloaded from Uniprot (ftp.uniprot.org). KEGG pathway information was assigned to all transcripts using the KAAS – KEGG Automatic Annotation Server (http://www.genome.jp/tools/kaas/)[41]. Gene ontology information was assigned based on sequence similarity with *Arabidopsis* using the Blast2Go server (http://www.blast2go.org). Protein domain scanning was performed using the 32,273 HMM models contained in the PFAM A/B databases [42] and the hmmer tools [43]. Annotation data was processed and integrated into the final transcriptome reference using custom Perl scripts and UNIX tools. Transcript identifiers were generated from a concatenation of the species initials (Pq), the Trinity component and subcomponent identifier numbers, followed by a period and splice variant number.

### Expression profiling and visualization

PCR duplicates were removed from filtered reads for each stage using Samtools before mapping reads against the assembled reference transcriptome using BWA (Q > 30). Reads were permitted to map to multiple places but only a single mapping using in downstream analysis. Investigation revealed that, presumably due to the very long read lengths, the vast majority of multiply mapped reads mapped to isoforms of the same gene. Reads with a map quality > 20 were pulled and counted for each transcript using Samtools [44]. The reads per kilobase of transcript per million reads mapped (RPKM) value was then calculated for each transcript in each developmental stage using R (Additional file 5). Relative distance between RPKM values was assessed using Pearson correlation coefficients (PCC) and the transcript distance matrix clustered using divisive hierarchical clustering before visualization in a heat map that scaled RPKM expression values row-wise to a mean of zero and standard deviation of one using a Z-score. Co-expression between individual transcripts was assessed using PCC between RPKM values across all seven stages of development sampled.

### Real time PCR analysis

After digestion with DNase I, approximately 1 μg of total RNA from stage 5 ripe fruit, stage 6 fruit drop and stage 7 senescence were converted into first-strand cDNA via the reverse-transcription reaction with random hexamer primers (New England Biolabs) and SuperScript III Reverse Transcriptase Kit (Invitrogen). The cDNA products were then diluted 10-fold with nuclease free deionized water before use as a template in real-time PCR. Specific cDNAs were amplified by SsoFast EvaGreen Supermix (Bio-Rad) in a volume of 10 ul. The reaction mixture contained 5ul SsoFast EvaGreen Supermix, 5 uM each of the forward and reverse primers, and 4 μl of template cDNA. PCR amplification was performed at the annealing temperature of 60°C with the CFX96 Real-Time PCR Detection System (Bio-Rad) according to the manufacturer’s instructions. Relative transcript abundances were calculated by the comparative cycle threshold method with GAPDH as an internal standard, using the Bio-Rad CFX Manager software (Version 3). Primer pairs for RT-PCR were designed based on online software (http://www.ncbi.nlm.nih.gov/tools/primer-blast/) and are listed in Additional file 4.

## Competing interests

The authors declare that they have no competing interests.

## Authors’ contributions

DW performed sample collection, RNA extraction, RT PCR, data analysis and drafted the manuscript. RA supervised DW in bioinformatic work, performed bioinformatic analysis of the data and helped write and edit the manuscript; SZ helped with plant preparation and sample collection; DB conceived the study, supervised DW and SZ, coordinated the project design, and edited the manuscript. All authors read and approved the final manuscript.

## Supplementary Material

Additional file 1 Growth chamber condition settings for plant material. Click here for file

Additional file 2 Quality score distribution of reads from all stages. Click here for file

Additional file 3 ***Panax quinquefolius *annotated transcriptome in Fasta format.** Click here for file

Additional file 4 Primer pairs for RT-PCR analysis. Click here for file

Additional file 5 RPKM values for all transcripts in transcriptome across seven stages. Click here for file
