# Supplementary material for: The root transcriptome for North American ginseng assembled and profiled across seasonal development
Source: BMC Genomics. 2013 Aug 19;14:564. doi: 10.1186/1471-2164-14-564 (PMC3751939; doi:10.1186/1471-2164-14-564)
Supplement: Additional file 1 — Growth chamber condition settings for plant material. [file 1471-2164-14-564-S1.docx]

**Additional file 1: Table S5: Growth chamber conditions**

| Date | Day temperature ºC | Night temperature ºC | Room humidity (%) | Light intensity  (umol/sqm/s) | Photoperiod  (light hours) |
| --- | --- | --- | --- | --- | --- |
| Feb 1-Mar 6 | 18 | 6 | 75 | 120 | 15.75 |
| Mar 7-Apr 6 | 24 | 12 | 75 | 120 | 15.3 |
| Apr 7-May 6 | 25 | 14 | 75 | 120 | 15 |
| May 7-Jun 6 | 26 | 13 | 75 | 120 | 14 |
| Jun 7-Jul 6 | 22 | 8 | 75 | 120 | 12.5 |
| Jul 7-Aug 6 | 13 | 3 | 75 | 120 | 11 |
| Aug 7-Sep 6 | 7 | -1 | 75 | 120 | 10 |
