# Supplementary material for: The root transcriptome for North American ginseng assembled and profiled across seasonal development
Source: BMC Genomics. 2013 Aug 19;14:564. doi: 10.1186/1471-2164-14-564 (PMC3751939; doi:10.1186/1471-2164-14-564)
Supplement: Additional file 2 — Quality score distribution of reads from all stages. [file 1471-2164-14-564-S2.pdf]

FastQC plots of Stage 1 read quality scores

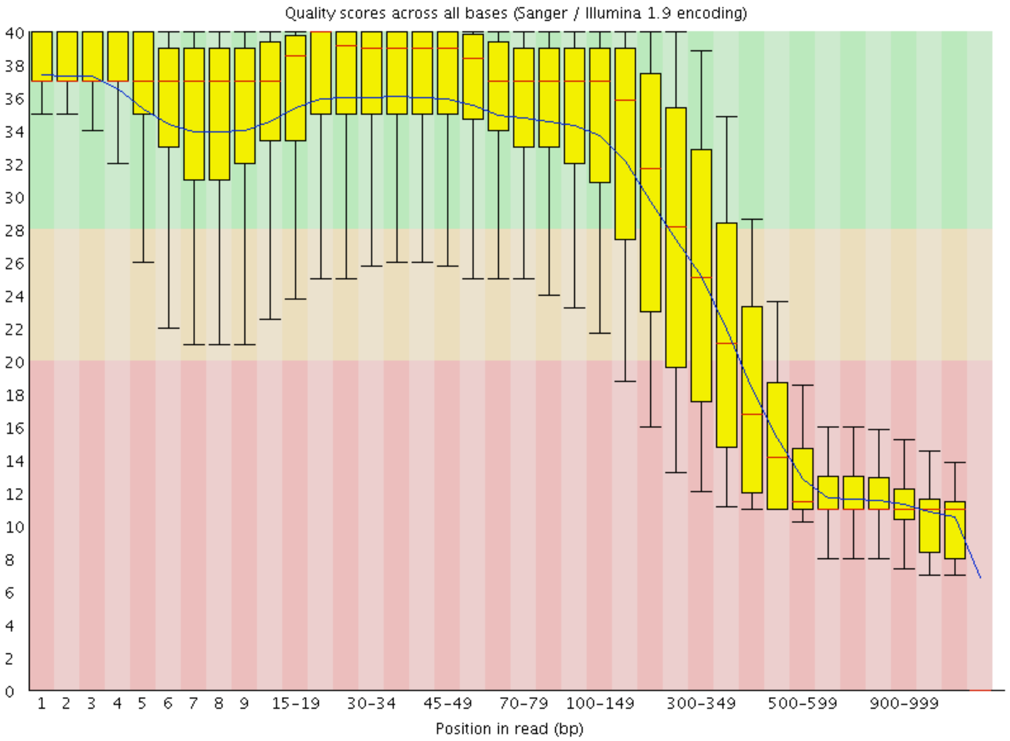

raw reads

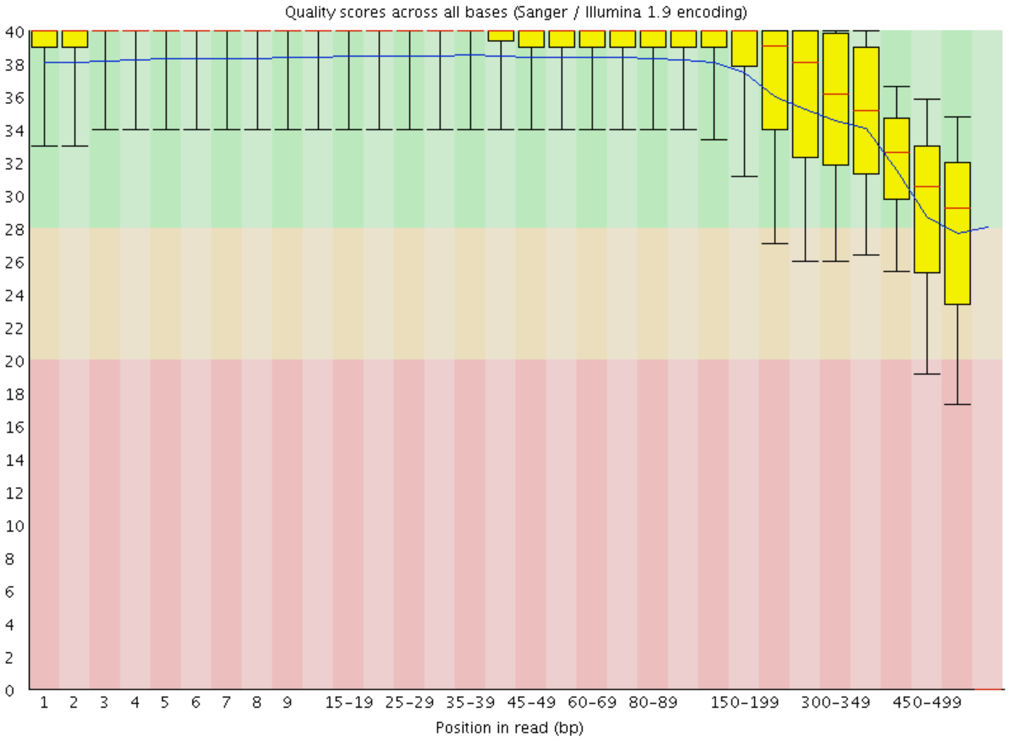

quality filtered reads

FastQC plots of Stage 2 read quality scores

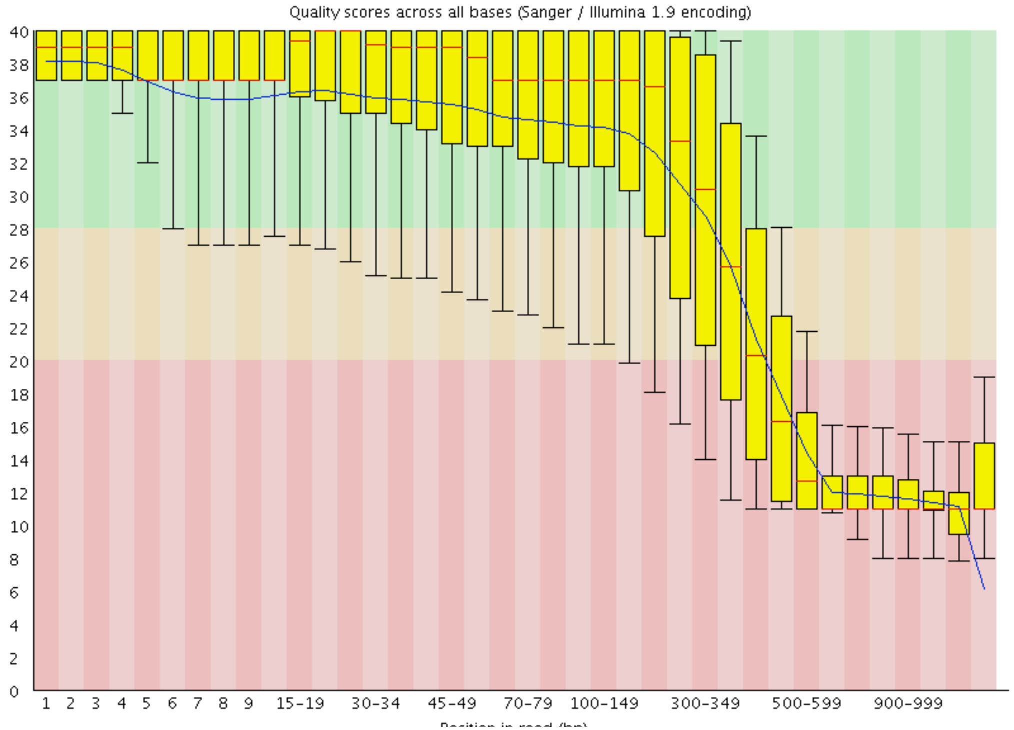

raw reads

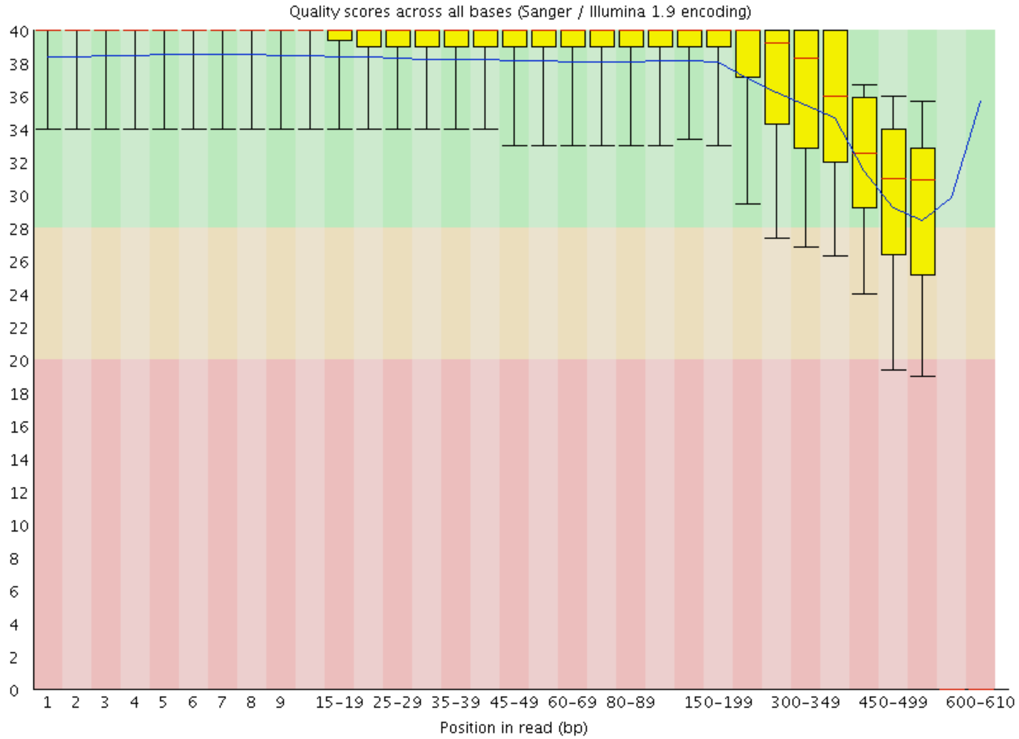

quality filtered reads

FastQC plots of Stage 3 read quality scores

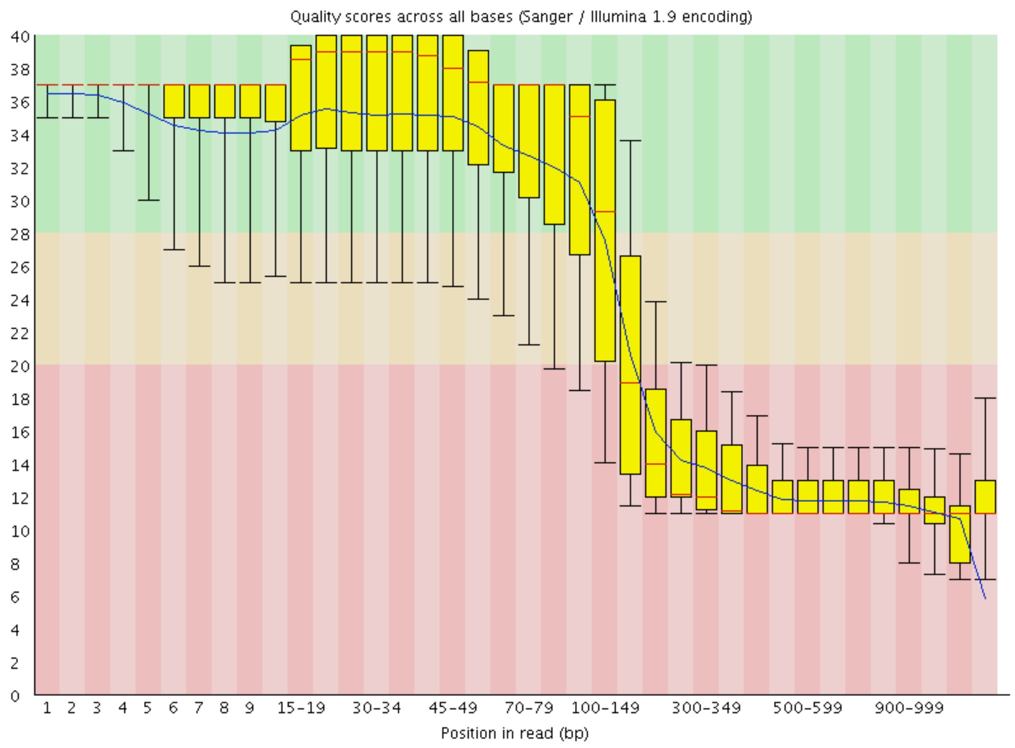

raw reads

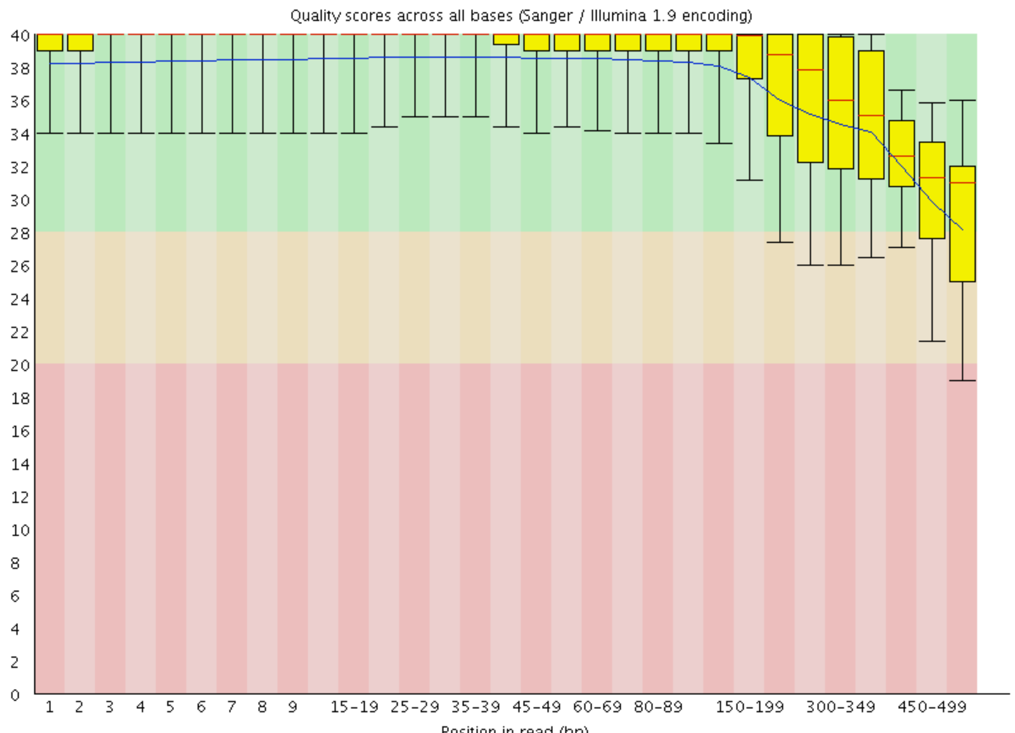

quality filtered reads

FastQC plots of Stage 4 read quality scores

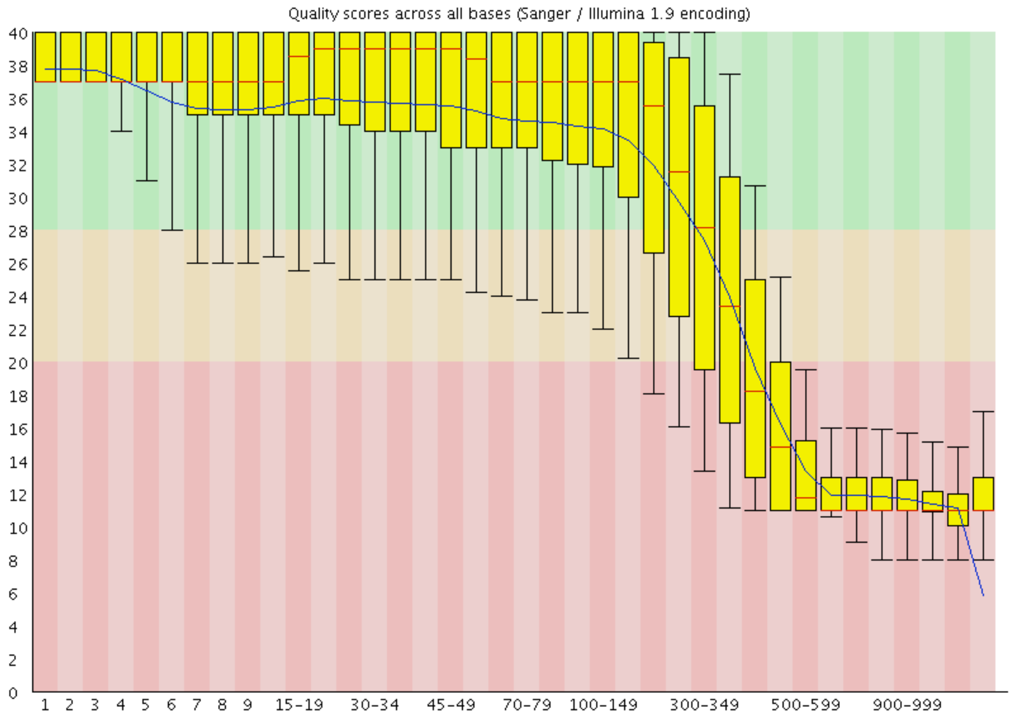

raw reads

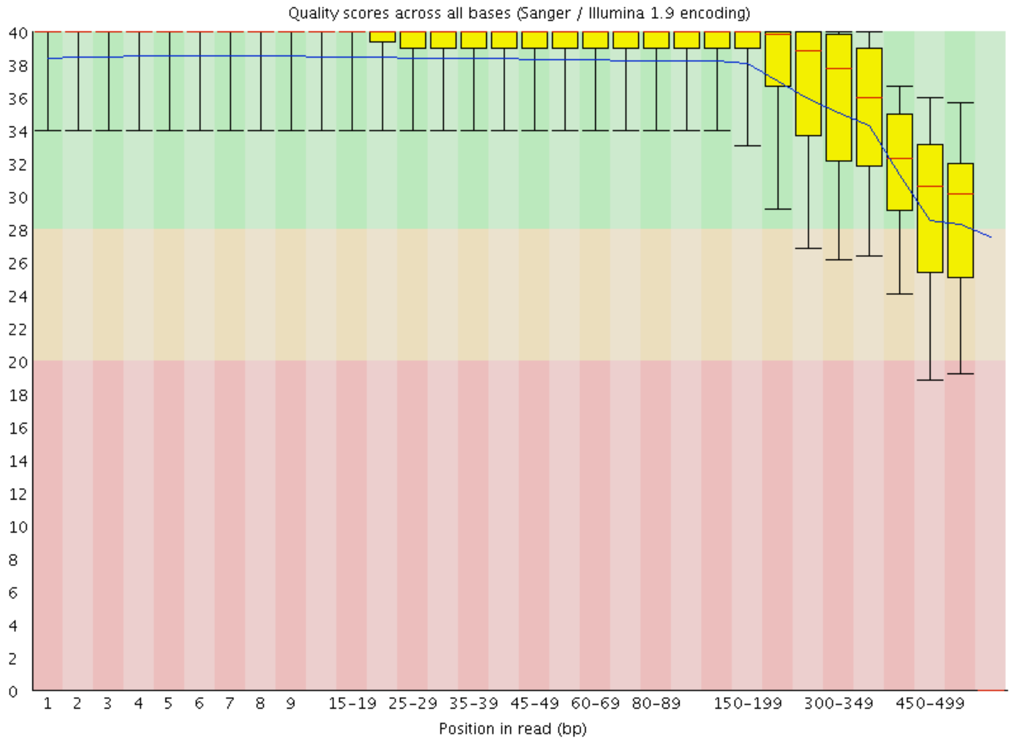

quality filtered reads

FastQC plots of Stage 5 read quality scores

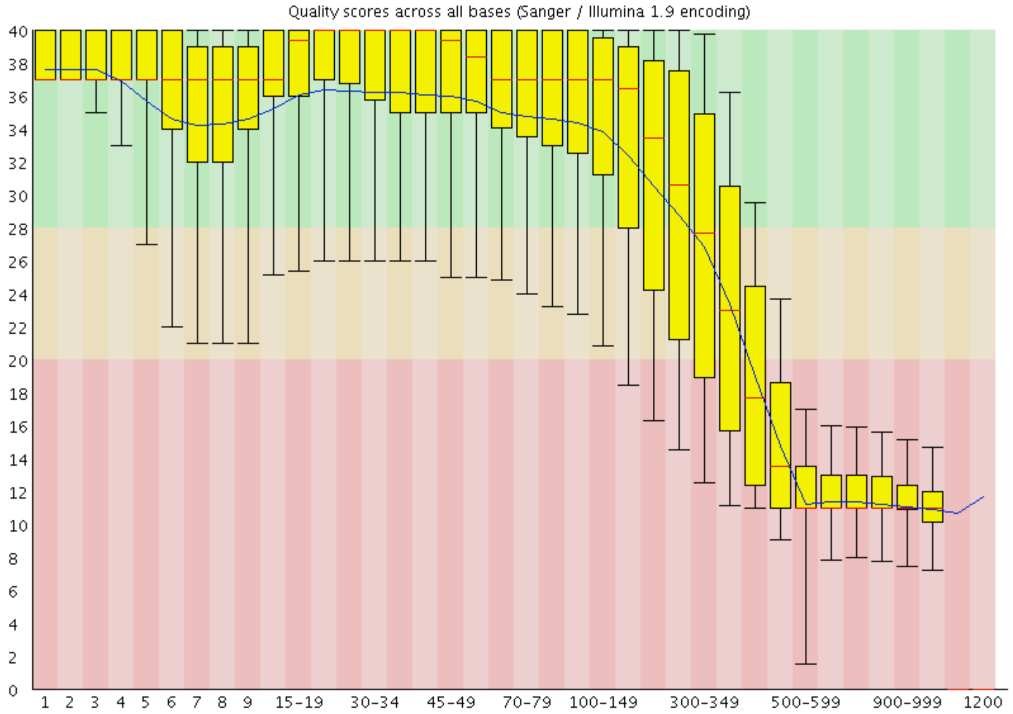

raw reads

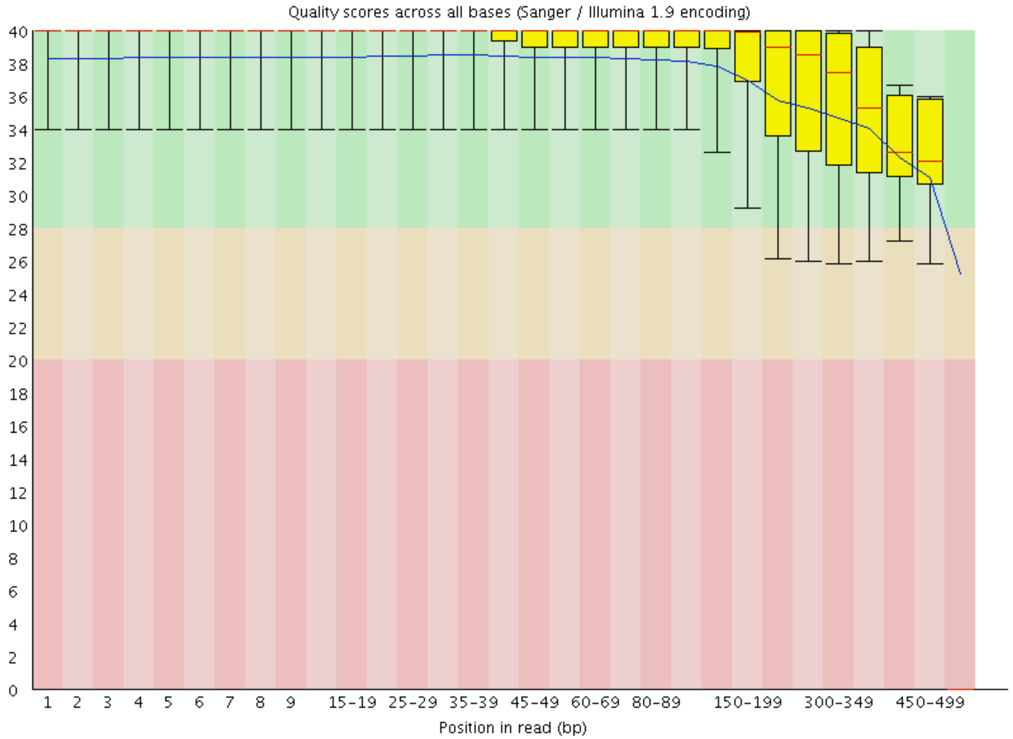

quality filtered reads

FastQC plots of Stage 6 read quality scores

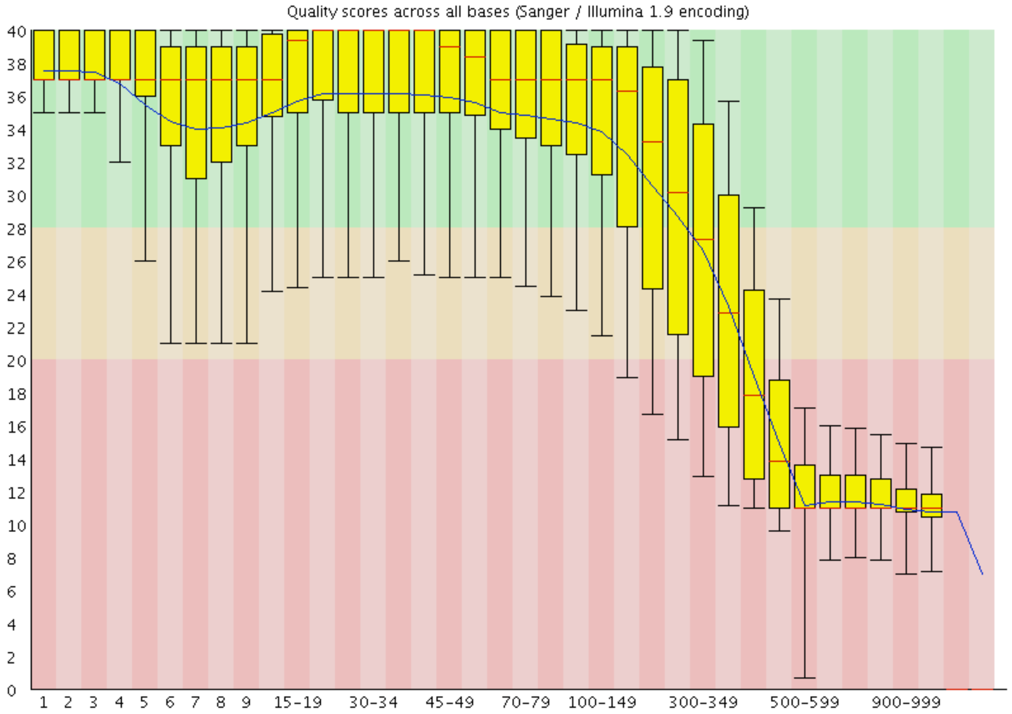

raw reads

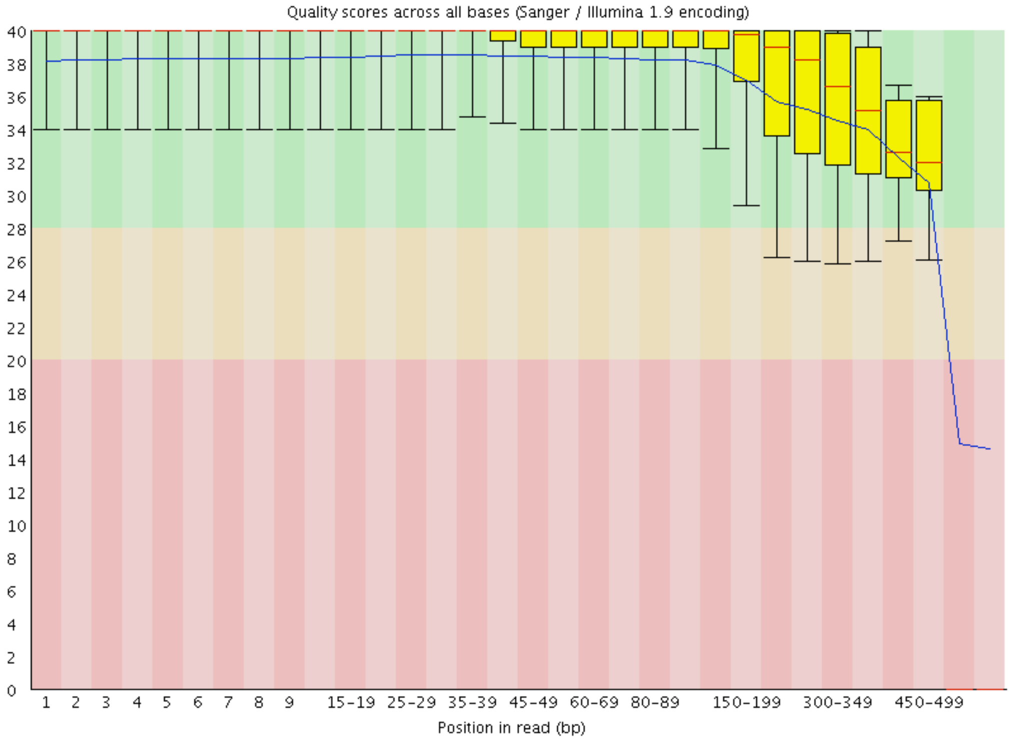

quality filtered reads

FastQC plots of Stage 7 read quality scores

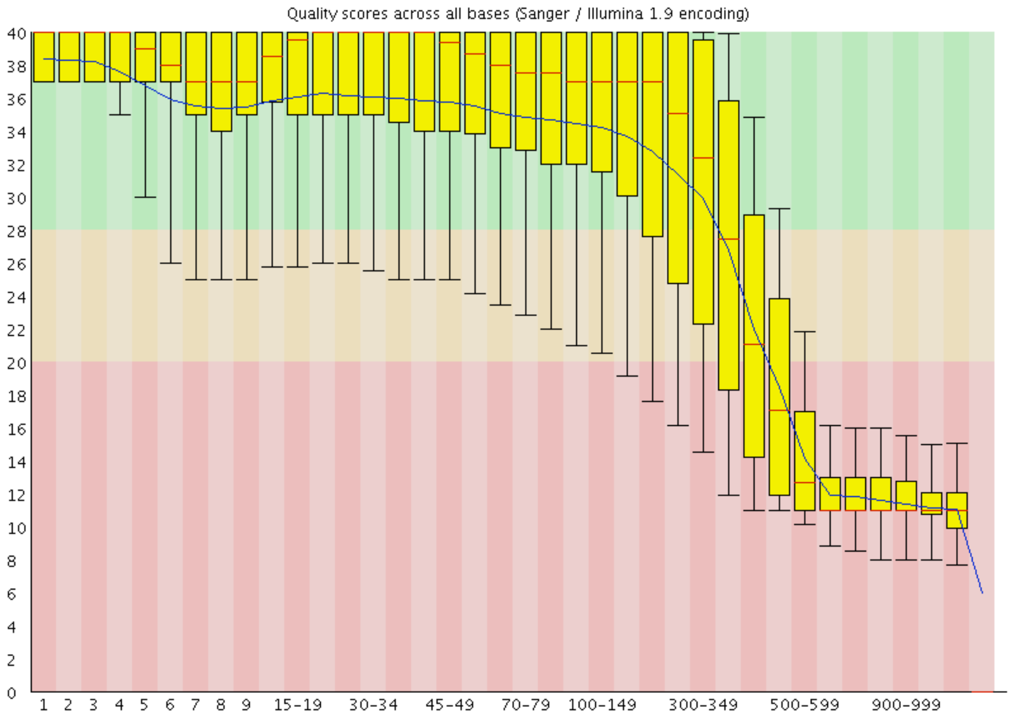

raw reads

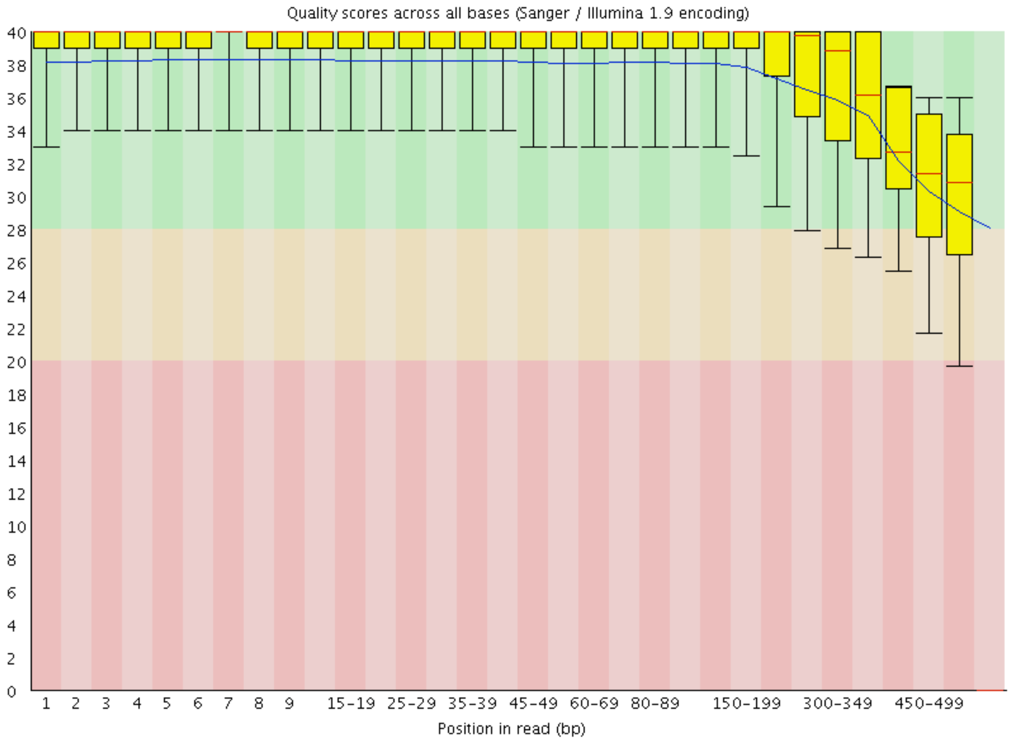

quality filtered reads
